# Ceramic Identity Contributes to Mechanical Properties and Osteoblast Behavior on Macroporous Composite Scaffolds 

**DOI:** 10.3390/jfb3020382

**Published:** 2012-05-23

**Authors:** Diana G. Morales-Hernandez, Damian C. Genetos, David M. Working, Kaitlin C. Murphy, J. Kent Leach

**Affiliations:** 1 Department of Biomedical Engineering, University of California, Davis, CA 95616, USA; Email: dgmorales@ucdavis.edu (D.G.M.); workingdm@gmail.com (D.M.W.); kmurphy@ucdavis.edu (K.C.M.); 2 Department of Anatomy, Physiology, and Cell Biology, School of Veterinary Medicine, University of California, Davis, CA 95616, USA; Email: dgenetos@ucdavis.edu; 3 Department of Orthopaedic Surgery, School of Medicine, University of California, Davis, Sacramento, CA 95817, USA

**Keywords:** bioceramic, hydroxyapatite, bioactive glass, composite, scaffold, bone

## Abstract

Implants formed of metals, bioceramics, or polymers may provide an alternative to autografts for treating large bone defects. However, limitations to each material motivate the examination of composites to capitalize on the beneficial aspects of individual components and to address the need for conferring bioactive behavior to the polymer matrix. We hypothesized that the inclusion of different bioceramics in a ceramic-polymer composite would alter the physical properties of the implant and the cellular osteogenic response. To test this, composite scaffolds formed from poly(lactide-*co*-glycolide) (PLG) and either hydroxyapatite (HA), β-tricalcium phosphate (TCP), or bioactive glass (Bioglass 45S^®^, BG) were fabricated, and the physical properties of each scaffold were examined. We quantified cell proliferation by DNA content, osteogenic response of human osteoblasts (NHOsts) to composite scaffolds by alkaline phosphatase (ALP) activity, and changes in gene expression by qPCR. Compared to BG-PLG scaffolds, HA-PLG and TCP-PLG composite scaffolds possessed greater compressive moduli. NHOsts on BG-PLG substrates exhibited higher ALP activity than those on control, HA-, or TCP-PLG scaffolds after 21 days, and cells on composites exhibited a 3-fold increase in ALP activity between 7 and 21 days *versus* a minimal increase on control scaffolds. Compared to cells on PLG controls, *RUNX2* expression in NHOsts on composite scaffolds was lower at both 7 and 21 days, while expression of genes encoding for bone matrix proteins (*COL1A1* and *SPARC*) was higher on BG-PLG scaffolds at both time points. These data demonstrate the importance of selecting a ceramic when fabricating composites applied for bone healing.

## 1. Introduction

The treatment of slow or nonhealing bone fractures is a significant clinical problem. Implants formed of metals, bioceramics, polymers, and decellularized tissues are under investigation to reduce or eliminate the current limitations of the “gold standard” of autograft bone. However, each of these materials also presents challenges in their application including wear debris formation and stress shielding, inadequate porosity to allow cellular infiltration, inability to be resorbed, or undesirable inflammatory responses [1]. Moreover, with the exception of polymeric systems, significant challenges exist to tailor these implants for the specific defect requiring treatment.

Calcium phosphate ceramics and bioactive glasses share similarities between their surface composition and chemical structure and the mineral phase of bone, and demonstrate enhanced osteoconductivity under *in vivo* settings [2]. Hydroxyapatite (HA; Ca_10_(PO_4_)_6_(OH)_2_) is the major mineral component of bone and is widely used as a bone substitute, both as a homogeneous implant and as a component of composite materials [3]. Βeta-tricalcium phosphate (β-TCP; Ca_3_(PO_4_)_2_) shares similar chemical composition as HA, but resorbs faster due to its lower Ca/P ratio and is weaker than HA per unit mass, making its use in treating defects in load-bearing bones more challenging [4]. Bioactive glasses (BG) exhibit tissue stimulatory properties and are under extensive investigation for their potential use in engineering of hard tissues [5,6]. However, the formation of implants which are wholly composed of bioceramics requires high temperatures, controlled cooling, and the resulting materials are brittle and slowly resorbable [7]. 

The development of matrices that possess sufficient strength, osteoconductivity, porosity, and degradation times represents a major focus in the arena of bone repair. Polymers derived from synthetic materials are commonly biocompatible, bioresorbable, and tailorable materials that can be molded into highly porous scaffolds [8]. For example, poly(lactide-*co*-glycolide) (PLG) is a commonly-used polymer for bridging bone tissue defects due to the ease of tailoring the degradation time by modulating the ratio of lactide and glycolide monomers during synthesis [9,10]. However, scaffolds formed of PLG lack sufficient mechanical strength for withstanding load, desirable osteoconductivity for integration with surrounding bone, or fail to provide instructional cues to the resident cells [11]. Moreover, there is a pressing need for conferring bioactive behavior to the polymer matrix.

In response to limitations of implants formed solely of bioceramics and polymers, we and others have developed composite scaffolds to capitalize on the beneficial aspects of the individual components. Polymers reinforced with bioceramics, fabricated using a variety of methods, consistently demonstrate improved mechanical properties over polymeric substrates without the constraints required of producing 3D bioceramic implants. The osteoconductivity and osteogenic potential of polymers is increased upon the addition of ceramics such as bone-like mineral, HA, TCP, and BG [12,13,14,15,16]. Composite scaffolds can have profound effects on other aspects of bone repair beyond inducing osteogenic differentiation. For example, we demonstrated that scaffolds containing nanosized HA enhance the osteogenic response and upregulate the secretion of potent proangiogenic trophic factors from human mesenchymal stem cells, thereby increasing the persistence of implanted cells, accelerating neovascularization, and enhancing bone formation [17,18]. BG stimulates angiogenesis *in vivo* [6,19], and vessel density and the quality of new bone formation was increased in calvarial defects treated with BG-coated scaffolds [20].

Despite significant evidence demonstrating the efficacy of bioceramic-polymer composite scaffolds for bone formation, little is known regarding potential differences in osteogenesis using composite scaffolds containing differing bioceramic particulates. We hypothesized that the identity of the ceramic incorporated within macroporous polymer scaffold composites would contribute to its material properties and osteogenic potential. In this study, composite scaffolds were fabricated using three common calcium phosphate materials: HA, TCP, or BG. We report that the addition of any bioceramic increases scaffold stiffness, decreases porosity, and differentially directs osteogenesis of NHOsts, with BG-loaded scaffolds potently stimulating osteogenesis compared to scaffolds containing other bioceramics. 

## 2. Experimental Section

### 2.1. Scaffold Preparation

Scaffolds were prepared using a gas foaming/particulate leaching method as described [18,21]. Briefly, PLG microspheres (85:15 DLG 7E; Lakeshore Biomaterials, Birmingham, AL, USA) were prepared using a double emulsion process. Bioceramic particulate, lyophilized microspheres, and NaCl particles (250–425 µm in diameter) were mixed in a 2.5:1:19 ratio, while control scaffolds were prepared without bioceramic. The selection of mass ratio was derived from our previous studies demonstrating increased stiffness while maintaining porosity in macroporous scaffolds at this mass ratio [18]. Composite scaffolds were fabricated using hydroxyapatite (HA, 100 nm diameter; Berkeley Advanced Biomaterials, Berkeley, CA, USA), β-tricalcium phosphate (TCP, <200 nm particle size, Sigma Aldrich, St. Louis, MO, USA), or 45S5 Bioglass^®^ (BG, 90–170 μm particle size, Novabone, Alachua, FL, USA). Mixtures were compressed in a stainless steel die using a Carver Press (Fred S. Carver) at 10 MPa for 1 min to produce solid disks (8.5 mm diameter, 1.5 mm thick). Disks were then placed under high pressure CO_2_ gas (5.5 MPa) for 16 h, after which the pressure was rapidly released to ambient to achieve polymer fusion. Solid disks were leached in distilled water for 24 h to remove the NaCl particles and generate highly porous scaffolds. 

### 2.2. Scaffold Characterization

Gross morphology of scaffolds was determined by scanning electron microscope images. Scaffolds were gold-coated using a sputter coater (Desk II; Denton Vacuum, Moorestown, NJ, USA). Specimens were imaged with a Hitachi S3500-N Scanning Electron Microscope at 10 kV. Pore diameter was quantified from scanning electron microscope images by measuring the long-axis of 20–40 pores in each scaffold using NIH Image J. Scaffold porosity was determined using Archimedes’ method [18]. Scaffolds were submerged in 100% EtOH in a custom-made vacuum bottle for 5 min until all bubbles were removed from the pores. The weight of the scaffolds before and after immersion was recorded, and scaffold porosity was calculated. 

The distribution of bioceramic particulate throughout the scaffolds was grossly observed by adsorption of Trypan blue as described [18,22]. Scaffolds were exposed to a 0.4% (w/v) Trypan blue solution (Alfa Aesar, Ward Hill, MA, USA) for 10 s. Scaffolds were rinsed twice in distilled H_2_O and placed in 100% EtOH for 1 min. Scaffolds were sonicated for 5 s at 40% power in 100% EtOH to remove remaining unbound dye and rinsed in distilled H_2_O before drying and analysis. The efficiency of bioceramic incorporation was determined by measuring the mass of individual components and final scaffold mass after fabrication.

Scaffold stiffness was determined by measuring the compressive modulus using an Instron 5,800 Series Testing System (Norwood, CA, USA). Samples were compressed with a constant deformation rate of 1 mm/min. The compressive modulus was calculated from the first 5% of the strain [18].

### 2.3. Cell Culture

Normal human osteoblasts (NHOsts) were purchased from Lonza (Clonetics^®^, Walkersville, MD, USA). NHOsts were expanded in DMEM (Invitrogen, Carlsbad, CA, USA) containing 10% fetal bovine serum (FBS, JR Scientific, Woodland, CA, USA) and 1% penicillin/streptomycin (Mediatech, Manassas, VA, USA) in standard cell culture conditions (37 °C, 5% CO_2_, 21% O_2_). Experiments were performed with cells between passages 2–5.

### 2.4. Osteogenic Potential

Scaffolds were sterilized in 95% EtOH for 30 min, rinsed twice with sterile PBS over 30 min, and incubated in DMEM for 30 min prior to cell seeding. NHOsts were statically seeded at 7.5 × 10^6^ cells/cm^3^ and allowed to attach for 1 h before moving cell-seeded constructs into DMEM. On day 0 (24 h post cell seeding), media was exchanged for fresh DMEM containing osteogenic supplements composed of 10 mM β-glycerophosphate and 50 µg/mL ascorbate-2-phosphate. Scaffolds were maintained under standard cell culture conditions on an XYZ shaker to enhance transport within the 3D construct, and media was changed every 3–4 days.

Samples were collected and analyzed after 4 h to assess cell seeding efficiency, or after 7 or 21 days to determine osteogenic potential. Briefly, scaffolds were rinsed in PBS and minced with a razor blade, incubated in 1X passive lysis buffer (Promega, San Luis Obispo, CA, USA) at room temperature for 10 min, sonicated briefly, and centrifuged at 10,000 rpm for 5 min. The supernatant was assayed for alkaline phosphatase (ALP) activity by incubating with 50 mM *p*-nitrophenyl phosphate (PNPP) in an assay buffer (100 mM glycine, 1 mM MgCl_2_, pH = 10.5) at 37 °C [12,23]. Absorbance was measured at 405 nm and converted to ALP activity using the extinction coefficient for PNPP (1.85 × 10^4^ M^−1^·cm^−1^). DNA content was quantified from lysate using a Quant-iT PicoGreen dsDNA kit (Invitrogen). To determine cell distribution, scaffolds were seeded with NHOsts and cultured for 1 day, decalcified in Calci-Clear (National Diagnostics, Atlanta, GA, USA) for 3 days, and hemotoxylin and eosin staining was performed on paraffin-embedded sections at 5 µm thickness.

The expression of genes associated with osteogenesis was measured in NHOsts seeded on composite scaffolds using qPCR. Briefly, total RNA from scaffolds was collected using an RNeasy Mini kit (Qiagen, Valencia, CA, USA) at 7 and 21 days. Between 200–1,000 ng of total RNA was reverse-transcribed with Superscript II Reverse Transcriptase (Invitrogen). Quantitative PCR was performed using primers and probes for *RUNX2*, *COL1A1*, and *SPARC* (Applied Biosystems, Foster City, CA, USA) on a Mastercycler^®^ realplex2 (Eppendorf, Westbury, NY, USA). Amplification conditions were 50 °C for 2 min, 95 °C for 10 min, followed by 40 cycles at 95 °C for 15 s and 60° C for 1 min. Quantitative PCR results were normalized to *RPL13* transcript level to yield ΔC_t_. Fold change in expression was subsequently calculated using the formula 2^−∆Ct^ [24].

### 2.5. Statistical Analysis

Results are expressed as mean ± standard deviation (SD) of the mean, assuming normal distribution of data sets, with the exception of PCR data, which is expressed as mean ± standard error of the mean (SEM). Statistical analyses were performed between two groups using the Student’s t-test or between multiple groups using a one-way ANOVA with Student Newman-Keuls multiple comparison *post hoc* test in GraphPad Prism^®^ 5 analysis software (GraphPad Software, San Diego, CA, USA). Probability values (*p*) for significance were calculated; *p* < 0.05 was considered statistically significant.

## 3. Results and Discussion

### 3.1. Scaffold Characterization

**Figure 1:**
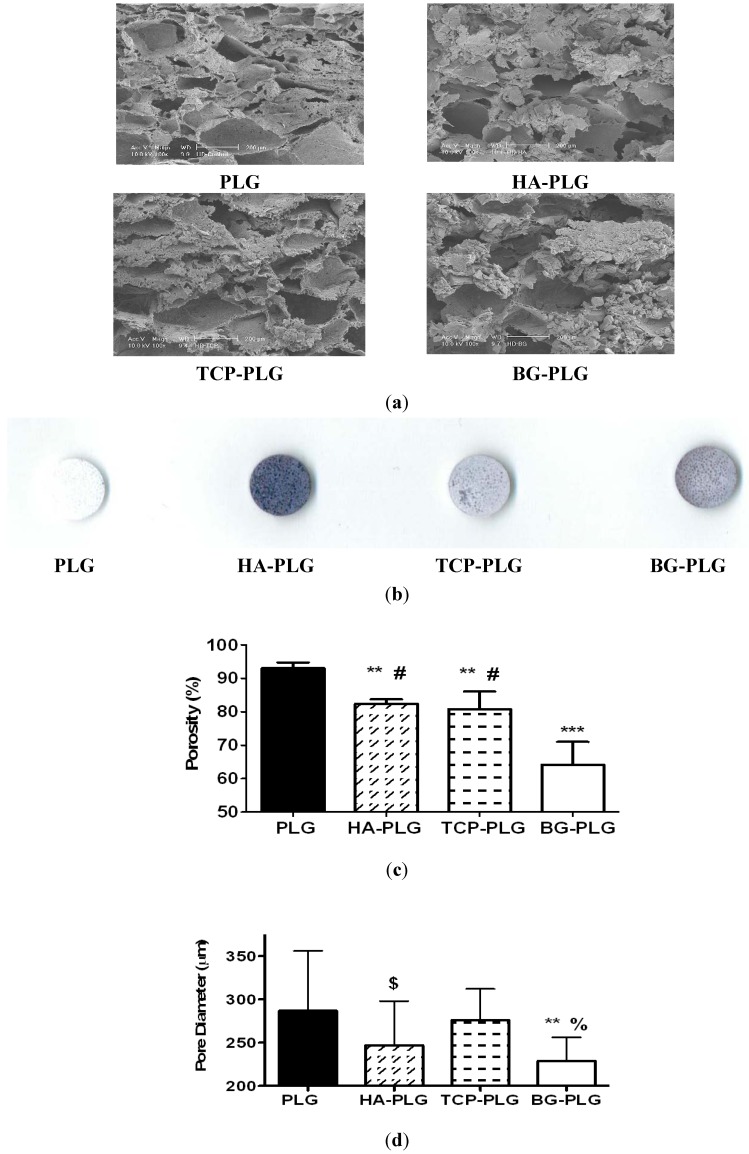
(**a**) Construct morphology was observed using scanning electron microscopy. Substrates imaged at 100X; scale bar represents 200 µm; (**b**) The presence and distribution of bioceramic was qualitatively observed by Trypan blue staining; (**c**) Scaffold porosity was determined using Archimedes’ method; (**d**) Scaffold pore diameter from each scaffold formulation. Data are mean ± SD (n = 5 for **a**–**c**; n = 20–40 for (**d**)). *** *p* < 0.0001 *vs.* PLG; ** *p* < 0.001 *vs.* PLG; $ *p* < 0.05 *vs.* PLG; # *p* < 0.0001 *vs.* HA-PLG and TCP-PLG; % *p* < 0.001 *vs.* TCP-PLG.

Bioceramic incorporation in macroporous scaffolds was highly efficient, as we did not detect any measurable loss in weight after scaffold fabrication for any group. We observed similar pore diameters for each scaffold type when imaging cross-sections using scanning electron microscopy (Figure 1a). The edges of the pores appeared rougher and less defined in composite scaffolds compared to PLG control scaffolds. We observed homogenous distribution of bioceramic particulate throughout the scaffolds when qualitatively assessed by Trypan blue adsorption (Figure 1b). HA-containing scaffolds adsorbed more dye than other composites. Scaffolds stained uniformly, despite differences in intensity for the stain, thus confirming homogenous distribution of the bioceramic throughout the composite scaffold. Composite scaffolds exhibited significant reductions in porosity compared to control scaffolds (93.0 ± 1.7%; Figure 1c). However, composites formed with HA and TCP had similar porosities (82.4 ± 1.3% and 80.8 ± 5.3%), respectively, while scaffolds containing BG showed reduced porosity (64.2 ± 6.8%). Moreover, scaffolds formed with BG exhibited lower pore diameters compared to other substrates (Figure 1d), suggesting that the ceramic was not entirely embedded within the polymer.

**Figure 2:**
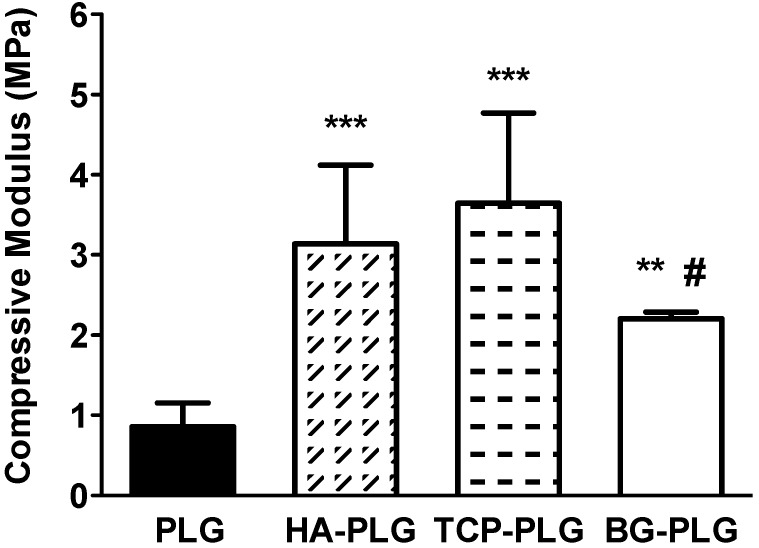
Influence of bioceramic on mechanical properties of substrate. Compressive modulus increased with the addition of bioceramic. Data are mean ± SD (n = 9 for PLG scaffolds, n = 5 for composite scaffolds). *** *p* < 0.0001 *vs.* PLG; ** *p* < 0.001 *vs.* PLG; # *p* < 0.05 *vs.* HA-PLG and TCP-PLG.

Compressive moduli increased upon the addition of any bioceramic. Composite scaffolds containing HA and TCP exhibited a 3–4 fold increase in compressive modulus compared to PLG scaffolds, while substrates containing BG exhibited a 2.5 fold greater compressive modulus, on average, *versus* control scaffolds (Figure 2). 

### 3.2. Osteoconductive Potential of Composite Scaffolds

**Figure 3:**
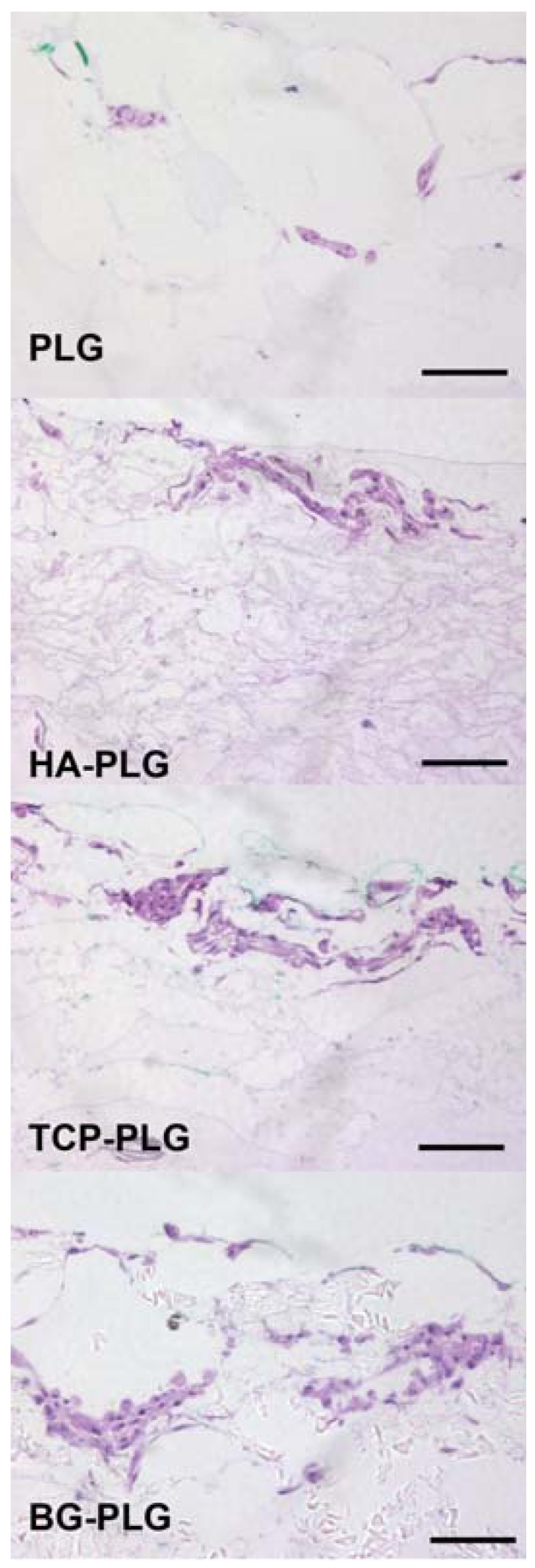
Distribution of cells on scaffolds observed by H&E staining. Images at 200× magnification (scale bar represents 100 µm).

**Figure 4:**
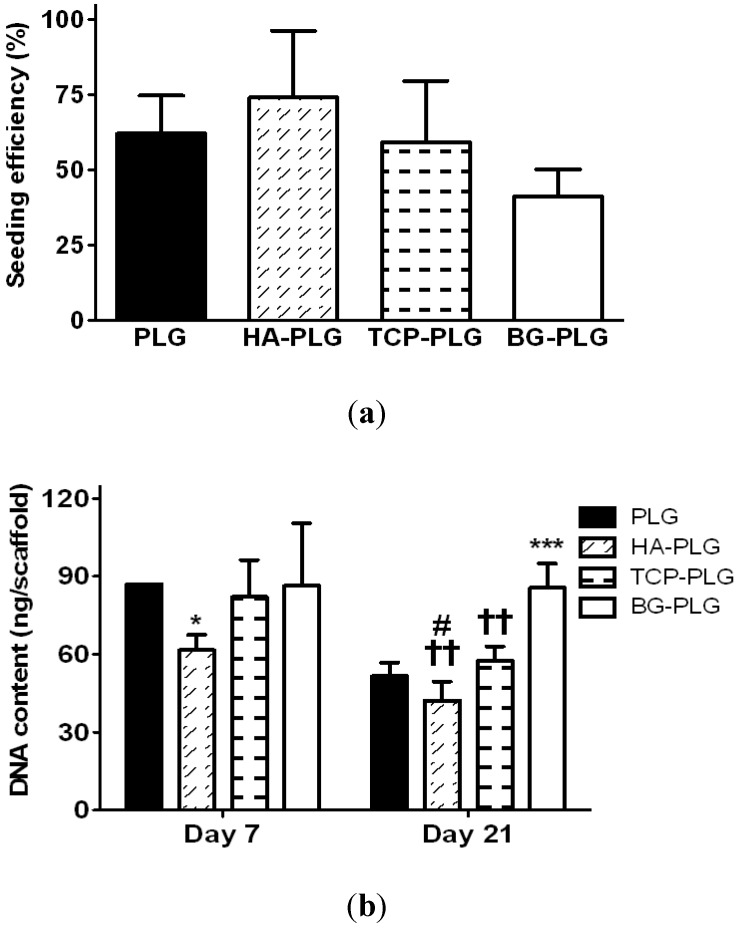
NHOst seeding efficiency (**a**) and proliferation (**b**) was measured by quantifying total DNA mass on 3D scaffolds. Data are mean ± SD (n = 4). * *p* < 0.05 *vs.* PLG control; *** *p* < 0.001 *vs.* PLG; †† *p* < 0.001 *vs.* BG-PLG; # *p* < 0.05 *vs.* TCP-PLG.

Upon examination by hemotoxylin and eosin staining, cells appeared to adhere predominantly to the outer surface of the scaffold (Figure 3). There were no statistically significant differences in cell seeding efficiency (Figure 4a). The ability of each scaffold to support cell proliferation and survival was assessed by quantifying DNA content on each scaffold after 7 or 21 days in culture. After 21 days, DNA content per scaffold was reduced in scaffolds containing PLG, HA, or TCP (Figure 4b). 

### 3.3. Osteogenic Response of NHOsts

**Figure 5:**
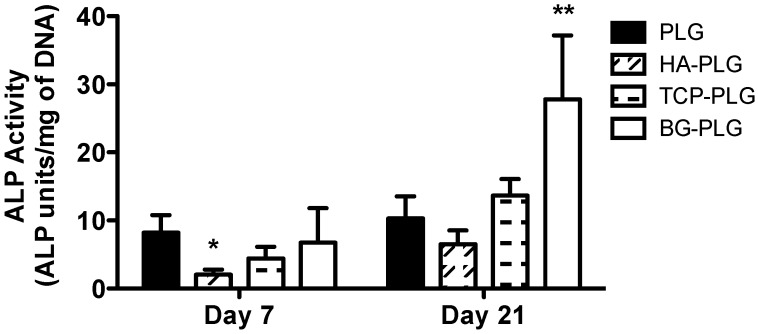
Alkaline phosphatase activity for NHOsts cultured on macroporous scaffolds. Data are mean ± SD (n = 4). *p < 0.05 *vs.* PLG at Day 7. **p < 0.01 *vs.* PLG, HA-PLG, and TCP-PLG at Day 21.

Changes in ALP activity were monitored as an indicator of osteoblastic differentiation of NHOsts as a function of scaffold composition (Figure 5). At Day 7, cells on HA-containing scaffolds exhibited significantly decreased ALP activity compared to PLG scaffolds, while cells on PLG, TCP-PLG, and BG-PLG exhibited statistically similar levels of enzymatic activity. After 3 weeks of culture, NHOsts on BG-PLG demonstrated significantly increased ALP activity compared to all other groups, while the remaining groups induced similar ALP activity. ALP activity increased over the 3-week culture period for all scaffolds. However, cells on composite scaffolds exhibited a comparable 3–4-fold increase in enzyme activity, while cells on control scaffolds demonstrated only a marginal increase over 21 days.

**Figure 6:**
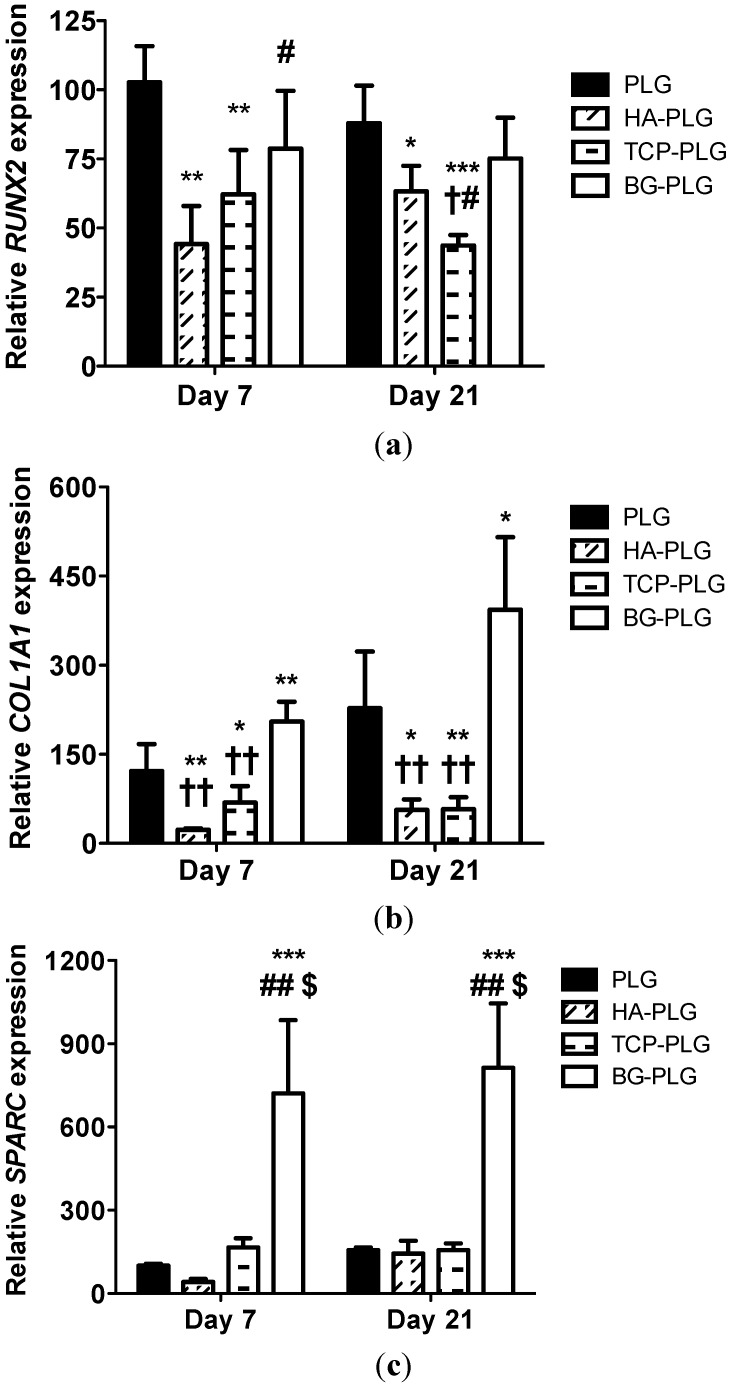
Quantitative PCR results for genes monitored in NHOsts over 3 weeks cultured on macroporous scaffolds: *RUNX2* (**a**), *COL1A1* (**b**), and *SPARC* (**c**). Values reflect fold change in the target mRNA expression over RPL13 *vs.* Day 7 PLG. Data are mean ± SEM (n = 4). * *p* < 0.05 *vs.* PLG control; ** *p* < 0.01 *vs.* PLG; *** *p* < 0.001 *vs.* PLG; # *p* < 0.05 *vs.* HA-PLG; ## *p* < 0.001 *vs.* HA-PLG; $ *p* < 0.001 *vs.* TCP-PLG; † *p* < 0.05 *vs.* BG-PLG; †† *p* < 0.001 *vs.* BG-PLG.

The expression of osteogenic marker genes *RUNX2*, *COL1A1*, and *SPARC* was analyzed by qPCR from 3D cultures after 7 or 21 days of culture. *RUNX2* is an obligate transcription factor for, and early indicator of, osteogenesis [25,26]. NHOsts on PLG control scaffolds exhibited greater *RUNX2* expression at both time points compared to cells on any composite scaffold (Figure 6a). Cells on HA- and TCP-containing scaffolds demonstrated significantly lower *RUNX2* expression than cells on control scaffolds at both time points. *RUNX2* expression of cells seeded on HA-PLG scaffolds was significantly lower than cells on BG composite scaffolds at Day 7, while cells on TCP scaffolds expressed lower *RUNX2* levels than those on BG composite scaffolds at Day 21.

Type I collagen is a major constituent of the organic matrix of bone, and *COL1A1* encodes for two of the three fibrils that compose collagen I filaments [27]. The influence of substrate composition on *COL1A1* transcript exhibited similar trends to *RUNX2* expression (Figure 6b). Specifically, cells on BG-PLG scaffolds demonstrated greater *COL1A1* expression *versus* all other composite scaffolds at Day 7, with cells on HA- and TCP-loaded scaffolds possessing significantly lower expression compared to PLG controls. After 21 days, NHOsts on BG-PLG scaffolds expressed significantly greater *COL1A1* transcript compared to the other composite substrates, and cells on HA- and TCP-containing scaffolds behaved similar to that of 7 days.

Osteonectin, encoded by the gene *SPARC*, is a bone-specific protein that is selectively bound to insolubilized type I collagen to enhance the complex binding of synthetic apatite crystals and free calcium ions, thus promoting the nucleation of mineral [28]. Thus, *SPARC* is a later marker of osteogenesis and signifies the onset of mature bone formation. *SPARC* gene expression profiles were similar to *COL1A1* and remained relatively constant in all groups throughout the study period (Figure 6c). NHOsts on BG-PLG scaffolds exhibited significantly higher *SPARC* expression at both 7 and 21 days, more than 4.3- and 5.2-fold, respectively, compared to remaining scaffolds. 

We sought to explore the contribution of individual bioceramics present within composite implants that are designed for applications in bone tissue engineering. Specifically, we aimed to determine if different ceramics would induce differences in physical properties when fabricating macroporous composite scaffolds, as well as the resulting osteogenic response of human osteoblasts. These data confirm that the resultant physical properties of composite scaffolds are dependent upon ceramic identity, while materials incorporated within osteoconductive bioceramic composite scaffolds differentially direct the behavior of normal human osteoblasts. 

Scaffolds that are designed for bridging bone defects and serving as successful cell carriers should ideally be highly porous in order to facilitate cellular invasion and host neovascularization, enable efficient transport of nutrients and waste removal to support cell survival, and promote integration with surrounding bone. The composition of such composite scaffolds is a critical mediator for material properties and cellular response. We fabricated scaffolds with a constant 2.5:1 mass ratio of ceramic to polymer based on our previously published studies that confirmed robust osteogenic response of human mesenchymal stem cells while maintaining high scaffold porosity [18]. Moreover, the compressive moduli of all composite scaffolds were within the lower limit of the compressive strength of trabecular bone (2–12 MPa) [29], while producing a scaffold with pore diameters large enough to enable vascularization and bone ingrowth [30]. By fixing the mass ratio of polymer, bioceramic, and porogen during the fabrication process, we discovered that the bioceramic identity strongly contributes to the resulting pore structure and porosity, likely due to interactions between the ceramic and polymer during the gas foaming/particulate leaching process. Pores of composite scaffolds exhibited a more irregular geometry than pores within control scaffolds. PLG control scaffolds were more than 90% porous, while the addition of HA and TCP reduced composite scaffold porosity to approximately 80%. The addition of BG further reduced scaffold porosity without visible differences in pore structure, suggesting that BG may occlude the micropores within the substrate, thus potentially limiting the success of this implant from the standpoint of diffusional transport to entrapped cells. Indeed, composite scaffolds containing BG possessed the smallest pore diameter, thus supporting the hypothesis that the ceramic is not fully embedded in the polymer. The decreased porosity of BG-PLG scaffolds may relate to the significantly increased particulate diameters used compared to HA and TCP. However, we aimed to fabricate composite scaffolds from commercially available bioceramics. Thus, the effect of nanosized BG incorporated into composite scaffolds on osteogenic response justifies further examination.

While scaffold porosity is an important factor relating to vascularization and nutrient transport, other factors may contribute to the success of the implant and the cellular response including substrate stiffness and exposure of ceramic from polymer. These two phenomena are likely linked due to the inherent hydrophilicity of most ceramic materials and hydrophobicity of most polymers. The incorporation of bioceramics into polymer composites increased the compressive strength of all materials. Although increases in substrate stiffness are commonly reported with ceramic-polymer composites, the relatively small increase in compressive modulus may be attributed to the lack of interfacial bonding strength between the ceramic phase and the polymer matrix [31]. Additionally, increases in substrate stiffness are likely due to a reduction in void space within the construct, as well as the ability of embedded material to support compressive load. In agreement with previous studies from our laboratory and others [14,18,32], we observed a corresponding increase in compressive modulus with decreasing porosity for both HA- and TCP-loaded scaffolds. However, BG-PLG scaffolds exhibited a lower compressive modulus than the other composite scaffolds while producing an even lower porosity. These data suggest that BG is not embedded within the polymer during gas foaming as effectively as the other bioceramics, and this may enhance osteogenesis by increasing the availability of osteostimulative ions resulting from BG dissolution to surrounding cells [33]. 

These data suggest that other factors beyond bulk mechanical properties may contribute to the cellular response. The exposure of ceramic resulting from differences in partitioning from the polymer, as well as ionic dissolution from the ceramic filler, may have a profound effect on the osteogenic potential of associated cells. Composites containing HA, which were previously coated with sucrose to minimize embedding in the polymer and thus maximize access and availability to surrounding cells, demonstrated significant increases in bone formation compared to scaffolds without sucrose-coated HA [34]. These data suggest that ceramic exposure is an important aspect to consider when designing these materials. Strategies to control interfacial bonding strength, perhaps by alkaline treatment to increase roughness and surface area [35], merit further investigation. Previous studies have reported the importance of the ionic dissolution products of BG to upregulate osteogenic gene expression in osteoblastic cells [36] or enhance local angiogenesis [23,37], and BG dissolves more rapidly than other bioceramics. Furthermore, the presence of different fillers may contribute to the degradation behavior of the ceramics and alter the local pH around the scaffold, hence contributing to the interaction of the material with the osteoblastic cells. 

Biomaterials used for matrix construction possess distinct affinities for plasma proteins, which contribute to cellular adhesion and construct integration with surrounding bone [38]. Like many other synthetic polymers, PLG is hydrophobic, and the incorporation of bioceramic produced more hydrophilic scaffolds. As shown in earlier work from our laboratory and others [18], nanosized HA was uniformly incorporated into porous scaffolds using the gas foaming process, and we observed similar distribution for TCP and BG. Previous studies report greater hydrophilicity of HA *versus* TCP when measuring contact angles of water on homogeneous ceramics [39]. Trypan blue staining demonstrated that composite scaffolds have a substantial portion of exposed bioceramic from the polymer, which may provide binding sites for plasma proteins or cells when implanted or used as a cell delivery vehicle. 

Osteoblasts play a critical role in the maintenance of mineral deposition and calcium-phosphate homeostasis. Bioceramics nucleate cell-secreted calcium and promote the formation of a mineralized microenvironment that directs subsequent osteoblast activity. Compared to osteoblasts on PLG control scaffolds, cells on HA- and TCP-PLG substrates exhibited lower expression of *RUNX2* and *COL1A1* at both time points. We have observed similar trends in osteogenic gene expression for human mesenchymal stem cells when cultured on PLG control substrates or PLG scaffolds coated with bone-like mineral, yet we observed increased ALP activity and calcium deposition [22]. This unexpected reduction may be due to a number of reasons. In the presence of increased concentrations of bone-like minerals, cells may alter their osteogenic program, thus shifting the temporal sequence of gene expression. Alternatively, proteins from the surrounding media may adsorb differentially to HA and TCP, thus initiating alternate integrin engagement and downstream signaling pathways that act alongside, or independent of *RUNX2* to modulate osteogenesis. In these studies, osteoblasts cultured on HA-PLG scaffolds demonstrated lower ALP activity and lower expression of *RUNX2*, *COL1A1*, and *SPARC*, signifying a less potent osteogenic response compared to other materials. Conversely, osteoblasts cultured on BG-PLG scaffolds uniformly exhibited a greater osteogenic response after 21 days of culture. BG degrades much faster than HA and TCP, and bioactive glasses activate numerous cellular pathways including osteogenic differentiation, cellular proliferation and metabolism by stimulating neighboring cells with their ionic degradation products [33]. In addition, 45S5^®^ Bioglass contains silica, a constituent that is lacking from the HA and TCP employed in this study. In previous studies characterizing the response of human osteoblasts seeded on silica surfaces without the interference of other ions present in glass ceramics, there were no apparent differences in cell number, metabolic activity, or ALP activity, yet nodule formation was accelerated on silica surfaces [40]. The importance of silica is confirmed in other studies demonstrating significant increases in ALP activity and type I collagen production by osteoblasts exposed to bioactive glasses with 46.1 mol% silica content (45S5) than cells exposed to bioactive glasses with 60 or 80 mol% silica (58S and 77S, respectively) [41].

## 4. Conclusions

The results of this study demonstrate that bioceramic selection plays an important role in the resulting biophysical properties and osteogenic potential of 3D composite scaffolds for use in bone tissue engineering. Using a fabrication process that avoids excessive heat or harsh organic solvents, we produced macroporous, biodegradable composite materials using three widely used bioceramics with compressive moduli on the order of trabecular bone and possessing osteogenic potential. Furthermore, these data suggest that the physical properties and osteogenic response can be further tailored by increasing polymer-ceramic interactions or through incorporating other materials such as bioactive glasses with increased silica content. These observations and principles may be valuable to tailor the properties of the implant to specific bone defects or develop alternative *in vitro* models of bone formation. 
